# bloodAGENT: a versatile tool for blood group typing and genomic variation analysis

**DOI:** 10.1093/bioadv/vbaf210

**Published:** 2025-09-02

**Authors:** Michael Wittig, Tim A Steiert, Christoph Gassner, Andre Franke

**Affiliations:** Institute of Clinical Molecular Biology, Christian-Albrechts-University and University Medical Center Schleswig-Holstein, Kiel 24105, Germany; Institute of Clinical Molecular Biology, Christian-Albrechts-University and University Medical Center Schleswig-Holstein, Kiel 24105, Germany; Institute of Clinical Molecular Biology, Christian-Albrechts-University and University Medical Center Schleswig-Holstein, Kiel 24105, Germany; Institute of Translational Medicine, Private University in the Principality of Liechtenstein, Triesen 9495, Liechtenstein; Institute of Clinical Molecular Biology, Christian-Albrechts-University and University Medical Center Schleswig-Holstein, Kiel 24105, Germany

## Abstract

**Motivation:**

Accurate blood group allele determination is essential for both research and clinical applications. While next-generation sequencing and third-generation sequencing technologies provide a wealth of genomic data, secondary analysis pipelines often struggle with detecting variations in paralogous regions and maintaining haplotype integrity. Existing tools for blood group allele determination are frequently proprietary and lack the flexibility needed to address these challenges. To bridge this gap, we developed bloodAGENT, a versatile and open-source tool designed for analyzing genomic variation and resolving blood group alleles.

**Results:**

bloodAGENT achieves high concordance in blood group allele determination under typical conditions but reveals a strong dependence on data completeness. Simulations show that dropout rates are the primary determinant of concordance and ambiguities, with noticeable declines at dropout rates as low as 5%. While phasing breaks have a minor overall impact, their importance remains evident in specific scenarios. These results highlight bloodAGENT’s robustness and its potential for handling complex genomic analyses.

**Availability and implementation:**

https://github.com/ikmb/bloodAGENT.git.

## 1 Introduction

Blood group systems are central to transfusion medicine, where accurate allele typing is essential to prevent alloimmunization. Beyond clinical diagnostics, blood group genotyping contributes to advances in population genetics, genomics, and personalized medicine (Ellinghaus *et al.* 2020, Castilho 2021). The increasing availability of Next-Generation Sequencing (NGS) and Third-Generation Sequencing (TGS) has enabled detailed genomic and haplotype-level analysis of blood group alleles (Steiert *et al.* 2022), revealing the complexity and diversity of these systems.

Several tools, such as RBCeq (Jadhao *et al.* 2022) and bloodTyper (Lane *et al.* 2018), have improved the accuracy of molecular blood group typing, particularly when benchmarked against serological data. However, these tools are closed-source and do not support fully phased TGS data, which limits transparency and the ability to resolve ambiguous allele combinations in diploid genomes.

To address these limitations, we developed bloodAGENT, the first fully open-source software for comprehensive blood group allele determination. It is designed to be modular, extensible, and transparent, encouraging community-driven development. bloodAGENT uses VCF and BigWig files from standard NGS/TGS pipelines and relies on ISBT-compliant annotation files to define allele- and haplotype-level configurations. The software applies a cosine similarity-based scoring function to match observed haplotypes with known references and supports phasing information when available, improving accuracy in cases with recombination or paralogous regions.

While optimized for blood group typing, bloodAGENT is adaptable to alternative genotyping platforms and broader genomic use cases. In this Application Note, we present the core functionality, performance under simulated and real-world conditions, and validation with serological and long-read reference datasets.

## 2 Methods

bloodAGENT is implemented in C++ with multithreading support for high-performance execution. It uses libhts (Bonfield *et al.* 2021) and libBigWig (Kent *et al.* 2010) to process standard input formats, VCF and BigWig. bloodAGENT includes Python scripts for result file parsing and for resolving secondary analysis issues that arise from standard tools. Configuration is defined via two ISBT-compliant files specifying variant mappings and haplotypes, supporting accurate allele-level typing across blood group systems (Karolchik *et al.* 2004, International Society of Blood Transfusion 2024).

The core algorithm applies cosine similarity (Han *et al.* 2011, Supplementary Methods, available as supplementary data at *Bioinformatics Advances* online) to compare observed variants to known haplotypes. When phasing information is available, it is used to reduce ambiguity in allele assignment. Output is returned as structured JSON (Bray 2017), facilitating integration into external tools and pipelines.

The software is cross-platform and runs on Linux and macOS; Singularity containers ensure portability and reproducibility across environments. The full source code, documentation, and example datasets are freely available at https://github.com/ikmb/bloodAGENT.

## 3 Results

**Figure 1.**
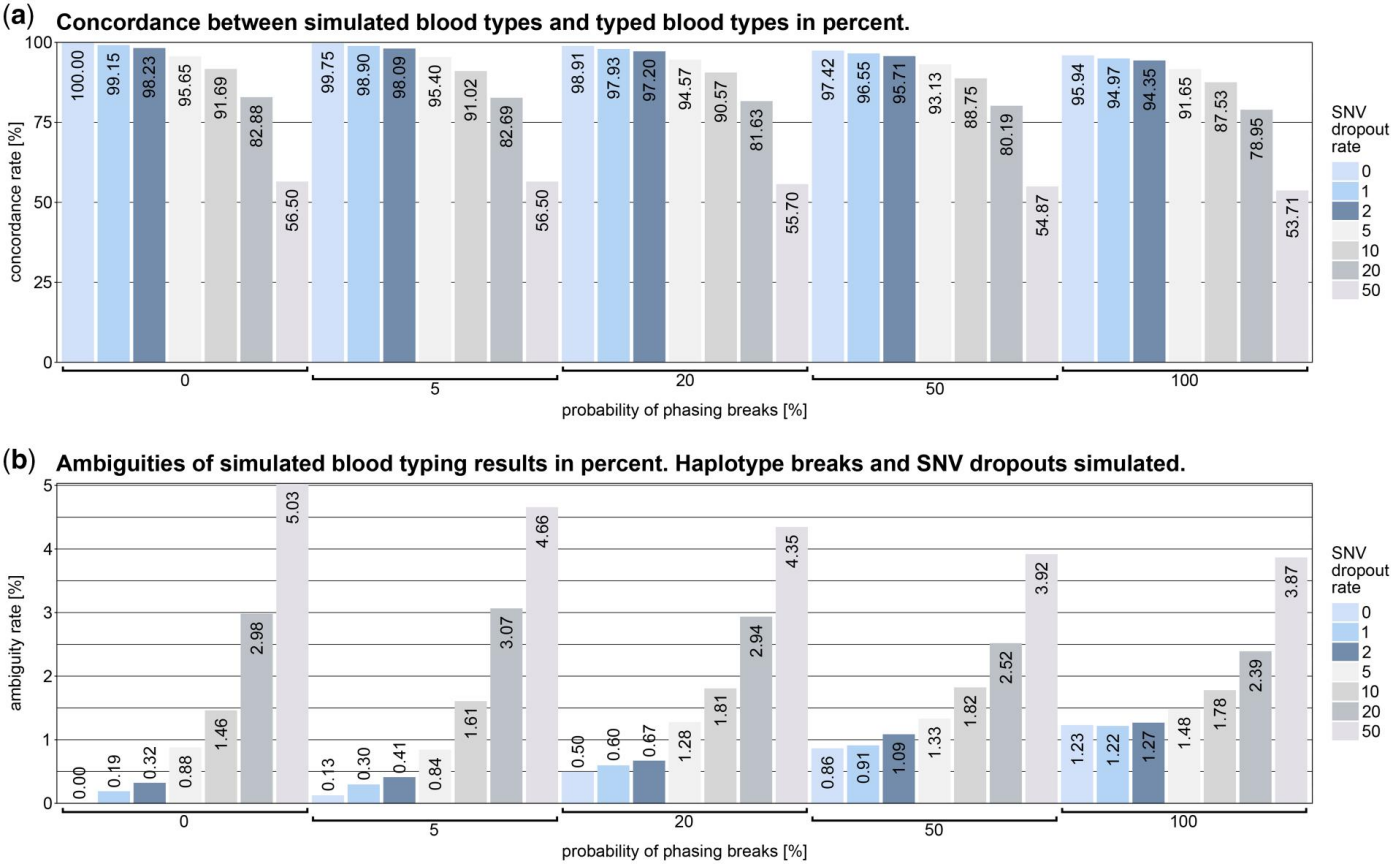
Concordance and ambiguities as a function of dropout and phasing break probabilities. (a) The concordance between simulated and predicted blood types decreases significantly with increasing dropout rates. At a dropout rate of 0%, the concordance remains at 100%, while at 50% dropout, accuracy falls below 50%. A noticeable decline begins at a dropout rate of 5%. Phasing breaks, in contrast, show minimal influence on concordance across all dropout rates. (b) The increase in ambiguities with rising error rates is depicted. Similar to concordance, dropout rates have the most pronounced effect on ambiguities, with higher dropout rates leading to substantially more ambiguous allele assignments. The influence of phasing breaks remains negligible in comparison.

To assess the robustness of bloodAGENT, we analyzed simulated datasets covering 56 combinations of dropout (0%–50%) and phasing break (0%–100%) rates, using 16 000 synthetic blood types per condition (Fig. 1). The analysis revealed that variant dropout is the dominant factor affecting allele call ambiguity and concordance. Phasing breaks contributed only minor additional effects. Concordance was 100% under perfect conditions and remained near that level for dropout rates below 5%, but dropped to approximately 55% under 50% dropout. These findings confirm the method’s stability under degraded input conditions.

For real-world validation, we analyzed 797 samples from the Human Genome Diversity Project (HGDP, Bergström *et al.* 2020) (Table 1, available as supplementary data at *Bioinformatics Advances* online), encompassing 16 blood group systems and 21 antigens. Initially unphased VCFs were phased with WhatsHap (Martin *et al.* 2016) to evaluate the benefit of haplotype information. Antigen-level concordance was assessed against reference genotypes obtained using the Hemo ID™ Blood Group Genotyping Panel for the MassARRAY^®^ system (Agena Bioscience), a MALDI-TOF-based assay routinely used in high-throughput blood group typing (Gassner *et al.* 2013, Table 5, available as supplementary data at *Bioinformatics Advances* online). Across all systems, antigen-level concordance reached 99.7% (Table 2, available as supplementary data at *Bioinformatics Advances* online). The ABO system demonstrated the most striking improvement: ambiguity dropped from 22.58% in unphased to 3.64% in phased data. This reduction stems from the resolution of frequent ambiguous allele combinations such as ABO*B.01/ABO*O.01.01 versus ABO*A1.01/ABO*O.01.41 (Fig. 1, available as supplementary data at *Bioinformatics Advances* online, Table 3, available as supplementary data at *Bioinformatics Advances* online). Other systems also benefited from phasing, though to a lesser degree.

To evaluate performance on phased long-read data, we analyzed samples from the GIAB collection (Zook *et al.* 2016) and four donors from the Institute of Transfusion Medicine (ITM) with matched serological data. bloodAGENT achieved 100% concordance with serological typing in ABO, RhDCE, and Kell systems. The RHCE C antigen, which could only be resolved at antigen level in short-read HGDP data, was successfully typed at allele level. These results underscore bloodAGENT’s applicability across platforms and confirm its capacity to handle complex loci when high-quality phasing is available.

Detailed genotype calls, ambiguity metrics, and concordance checks are available in Tables 2–10, available as supplementary data at *Bioinformatics Advances* online.

## 4 Discussion

The presented results underscore the robustness and adaptability of bloodAGENT in analyzing genomic data for blood group allele determination, even under varying conditions of data quality. The limited influence of phasing breaks on both concordance and ambiguities, as observed in the simulated datasets, highlights the dominant role of dropout rates in determining analysis accuracy. Nevertheless, haplotype phasing remains critical in specific scenarios. Examples such as the blood group systems ABO or FY illustrate the necessity of maintaining accurate phasing data to achieve reliable allele determination for specific blood group systems.

While simulations provide a robust baseline, real-world data introduces complexities that are difficult to model in silico. The secondary analysis pipelines used to generate input files, such as DRAGEN (Behera *et al.* 2025), GATK (McKenna *et al.* 2010), and deepVariant (Poplin *et al.* 2018), can vary in their ability to detect structural variations and small insertions/deletions. For example, the deletion of the *RHD* gene (RHD*01N.01) is rarely detected in short-read data, and the 109 bp insertion in *RHCE*, which defines RHCE*02/*04, is often missed by deepVariant but detected more reliably in GATK-TGS workflows. These limitations can affect the resolution of key antigen systems. While the C antigen in RHCE can be classified at antigen level via coverage heuristics (Lane *et al.* 2018), full allele-level typing requires long-read sequencing. Similarly, RHD+/- status can be inferred via coverage, too.

bloodAGENT addresses some of these challenges by allowing flexible data input. Users can supplement primary variant calls with additional VCFs derived from orthogonal pipelines or custom coverage analysis scripts provided with the software. This enables adaptation to system-specific limitations and improves call reliability in paralogous regions. Importantly, this design ensures long-term compatibility with future sequencing technologies, including TGS platforms with improved error correction and structural variant detection.

A limitation of our benchmark lies in the nature of the reference data, which relied on molecular typing via MALDI-TOF for all systems except ABO, where an alternative molecular method, microarray-based genotyping (Wittig *et al.* 2024), was used. Serology remains the gold standard. In this study, large-scale evaluation was based on molecular reference data (e.g. HGDP cohort), and only a small-scale serological benchmark was available using four ITM donor samples. Wider availability of curated serological data, especially in public datasets like 1000 Genomes (Abecasis *et al.* 2012), would significantly improve future validations.

The limitations of our reference dataset were particularly evident in the MNS system. The applied minor MALDI-TOF panel (Gassner *et al.* 2013, Meyer *et al.* 2016) is not designed for detecting the NULL alleles GYPB*03N.02, GYPB*03N.03, and GYPB*03N.04. In contrast, bloodAGENT identified null alleles such as GYPB*03N.02, GYPB*03N.03, and GYPB*03N.04, particularly among African samples. For consistency in benchmarking, we merged these alleles under GYPB*03 to align with the MALDI-TOF output. This approach preserved comparability while acknowledging that bloodAGENT’s resolution exceeds that of the reference method. A full breakdown of affected genotypes is provided in Tables 3 and 4, available as supplementary data at *Bioinformatics Advances* online.

Beyond tool development, our findings emphasize the need for greater transparency in blood group variant curation. We encourage the ISBT community to share detailed phasing and variant data, particularly for rare and hybrid alleles. Current ISBT classifications do not account for all possible haplotype combinations, and databases such as Erythrogene (Möller *et al.* 2016) list many variant combinations without ISBT labels despite comprising known ISBT variants only. Capturing, naming, and benchmarking such haplotypes would significantly advance the field.

In conclusion, bloodAGENT demonstrates robustness under degraded input conditions and flexibility across sequencing platforms. Its modular architecture and support for supplementary data integration make it well-suited for ongoing use in both research and clinical settings. Future advances in variant detection, particularly for structural variation, will further improve allele-level typing accuracy in blood group genetics.

## Supplementary Material

vbaf210_Supplementary_Data

## Data Availability

The data underlying this article was published by Bergström *et al.* (2020) and are available at https://ngs.sanger.ac.uk/production/hgdp/hgdp_wgs.20190516/. The software and test data are available at https://github.com/ikmb/bloodAGENT.
