# Supplementary material for: bloodAGENT: a versatile tool for blood group typing and genomic variation analysis
Source: Bioinform Adv. 2025 Sep 2;5(1):vbaf210. doi: 10.1093/bioadv/vbaf210 (PMC12448795; doi:10.1093/bioadv/vbaf210)
Supplement: vbaf210_Supplementary_Data [file vbaf210_supplementary_data.zip › bloodAgent_Bioinf_Application_Note_Supplementary_Methods.docx]

| Supplementary Methods (Workflow and Allele Assignment) of  bloodAGENT: A Versatile Tool for Blood Group Typing and Genomic Variation Analysis  Michael Wittig^1,*^, Tim A. Steiert^1^, Christoph Gassner^1,2^, Andre Franke^1^  ^1^ Institute of Clinical Molecular Biology, Christian-Albrechts-University and University Medical Center Schleswig-Holstein, Kiel 24105, Germany  ^2^ Institute of Translational Medicine, Private University in the Principality of Liechtenstein, Triesen 9495, Liechtenstein  *Corresponding author. Zentrum Molekulare Biowissenschaften, Am Botanischen Garten 11 – 24118 Kiel, Germany. E-mail:m.wittig@ikmb.uni-kiel.de |
| --- |

# Pipeline Overview

The bloodAGENT software processes phased and non-phased genomic variant data to predict blood group alleles based on ISBT-defined reference profiles. The workflow is modular by design and does not include upstream processing steps such as read alignment, variant calling, or phasing. These are expected to be performed externally using tools of the user's choice. In our validation, we employed standard pipelines including BWA for alignment, GATK for variant calling, and WhatsHap for phasing. While the choice of upstream tools remains flexible, we specifically opted for WhatsHap for phasing, as its read-backed phasing strategy directly reflects the sequencing evidence, allowing us to analyse the measured genomic structure without relying on statistical inference, which can be problematic in structurally complex and paralogous loci such as RHD/RHCE and MNS. All of the aforementioned secondary analysis tools, as well as others such as DeepVariant or Bowtie, provide detailed best-practice guidelines and workflow recommendations aimed at achieving optimal results.

# Reference Databases and Input Files

The software requires three reference files: (1) an ISBT-based SNP annotation table, (2) a curated ISBT allele definition table, and (3) a RefSeq-based gene annotation file. All reference files are included in the software package. Per sample, a phased VCF file and a BigWig coverage file must be provided. These two files are sample-specific and are typically generated as part of an NGS/TGS secondary analysis pipeline.

# Annotation and Filtering

Sample variants are first annotated against the ISBT SNP table and filtered to retain only positions relevant to blood group determination. These filtered variants form the basis for downstream allele resolution.

# Haplotype Construction and Scoring

For each blood group system, bloodAGENT derives candidate haplotypes from the phased variant data. In cases where the phase information is incomplete or disrupted, such as phasing breaks across distant variants, the software generates all possible haplotype combinations consistent with the observed phase blocks. Each candidate haplotype is then converted into a binary presence/absence vector that encodes the presence of allele-defining SNPs. In parallel, each known ISBT allele is also represented as a binary reference vector. To determine the best matching allele, the tool calculates cosine similarity between the sample haplotype vector and each reference allele vector. Cosine similarity is defined as:

$${cosine}_{similarity}\left( A,B \right)=\frac{A\cdot B}{\left\| A \right\|\left\| B \right\|}$$

where 𝐴 is the sample vector and B is the reference allele vector. This metric is especially well-suited for sparse or variable-length feature vectors, as it captures directional similarity rather than absolute distance. This enables robust matching even when a subset of expected variants is missing due to dropout, coverage gaps, or sequencing artifacts.

# Allele Assignment and Output

The allele(s) with the highest similarity scores are assigned per blood group system. Results are returned in a structured JSON output file containing allele predictions, associated confidence scores, and quality metrics. The system currently supports 40 blood group systems, based on 1230 ISBT variants and 1378 curated alleles.

# Upstream Configurations: Advantages and Limitations

The performance of bloodAGENT depends directly on the quality of its upstream input data. As the tool does not perform alignment, variant calling, or phasing itself, it relies entirely on the integrity of phased VCFs generated by standard NGS or TGS secondary analysis pipelines. Consequently, any limitations or biases introduced during these upstream steps will propagate into the allele prediction.

Read alignment is typically performed using short-read mappers such as BWA-MEM, which are effective in most genomic regions. However, they encounter limitations in loci with high sequence homology or structural complexity, such as the RHD/RHCE or GYP gene clusters. In these regions, incorrect or ambiguous mapping can result in either false variant calls or missing data. These mapping issues also affect phasing quality and can interrupt haplotype blocks that are essential for accurate allele resolution.

Variant calling using tools like GATK or DeepVariant delivers high accuracy for SNPs and small indels but does not capture structural variants such as gene deletions or duplications unless supplemented with dedicated CNV detection. This limitation is particularly relevant for blood group systems like RHD, where the presence or absence of an entire gene copy has phenotypic relevance. In cases of heterozygous gene deletions, standard variant callers may report all SNPs in the deleted region as homozygous for the present allele, while they are in fact hemizygous. This leads to misleading genotypes and misinterpretation during allele assignment.

Phasing is a critical step for bloodAGENT, as the tool operates on haplotype-level variant combinations. Statistical phasing approaches such as SHAPEIT5 (Hofmeister *et al.* 2023) are efficient for large datasets and work well for common alleles, but they rely on population priors and do not incorporate direct sequencing evidence. This can lead to incorrect phase assignments in rare alleles or across large genomic distances. Read-backed phasing tools like WhatsHap, in contrast, reconstruct haplotypes based on the actual sequencing reads, offering higher accuracy in single-sample settings and better performance in complex loci. However, they are limited by the insert size and read connectivity and may produce fragmented phase blocks, especially in low-coverage regions.

Across all upstream configurations, one central challenge remains: bloodAGENT can only work with what is present in the input VCF. Variants that are misaligned, filtered out, incorrectly phased, or simply missing due to sequencing or calling thresholds are not recoverable at the annotation stage. This is particularly evident in cases where variant dropout or poor phasing leads to ambiguous or incorrect allele predictions, as shown in Figure 1 of the main manuscript.

In summary, the choice of alignment, variant calling, and phasing tools has a substantial impact on the accuracy of bloodAGENT predictions. For systems with complex or paralogous gene structures, careful alignment and read-backed phasing are essential. The integration of CNV detection is also strongly recommended for systems where gene presence or dosage affects typing. While bloodAGENT is modular and remains agnostic to the upstream pipeline used, its predictive accuracy is intrinsically limited by the quality and completeness of the input data.

# Comparison of WhatsHap and SHAPEIT5 for Phasing in Blood Group Genotyping

In addition to reconstructing measurable haplotypes with WhatsHap, we also employed SHAPEIT5 to infer the most likely genotypes. In short-read sequencing data, WhatsHap often fails to produce haplotypes that span the full transcript, whereas SHAPEIT5 typically generates continuous haplotypes across the entire locus. While we consider the WhatsHap approach preferable when the data permit, particularly for long-read sequencing, where haplotypes can be directly observed rather than statistically inferred, SHAPEIT5 demonstrated notable advantages in short-read datasets.

In the ABO system specifically, SHAPEIT5 reduced phasing ambiguities, likely due to its ability to generate uninterrupted haplotype blocks from short-read data. For example, in HGDP00944, HGDP00630, HGDP00478, and HGDP00467, SHAPEIT5 assigned more common alleles and/or excluded unlikely rare variants compared to WhatsHap, supporting the SHAPEIT5 predictions. Conversely, in HGDP00757 and HGDP00721, the results from WhatsHap are more likely to be correct. Overall, our findings for ABO suggest that SHAPEIT5, when applied to short-read data, reduces ambiguity and often yields more consistent allele calls. This performance could likely be further improved by generating blood group–specific reference panels for SHAPEIT5.

Full details are provided in **Supplementary Methods Table SM1**. The trend observed for ABO extends to all other blood group systems examined, further reinforcing our conclusion that prediction quality is primarily determined by upstream processing, specifically read alignment, variant calling, and phasing.

**
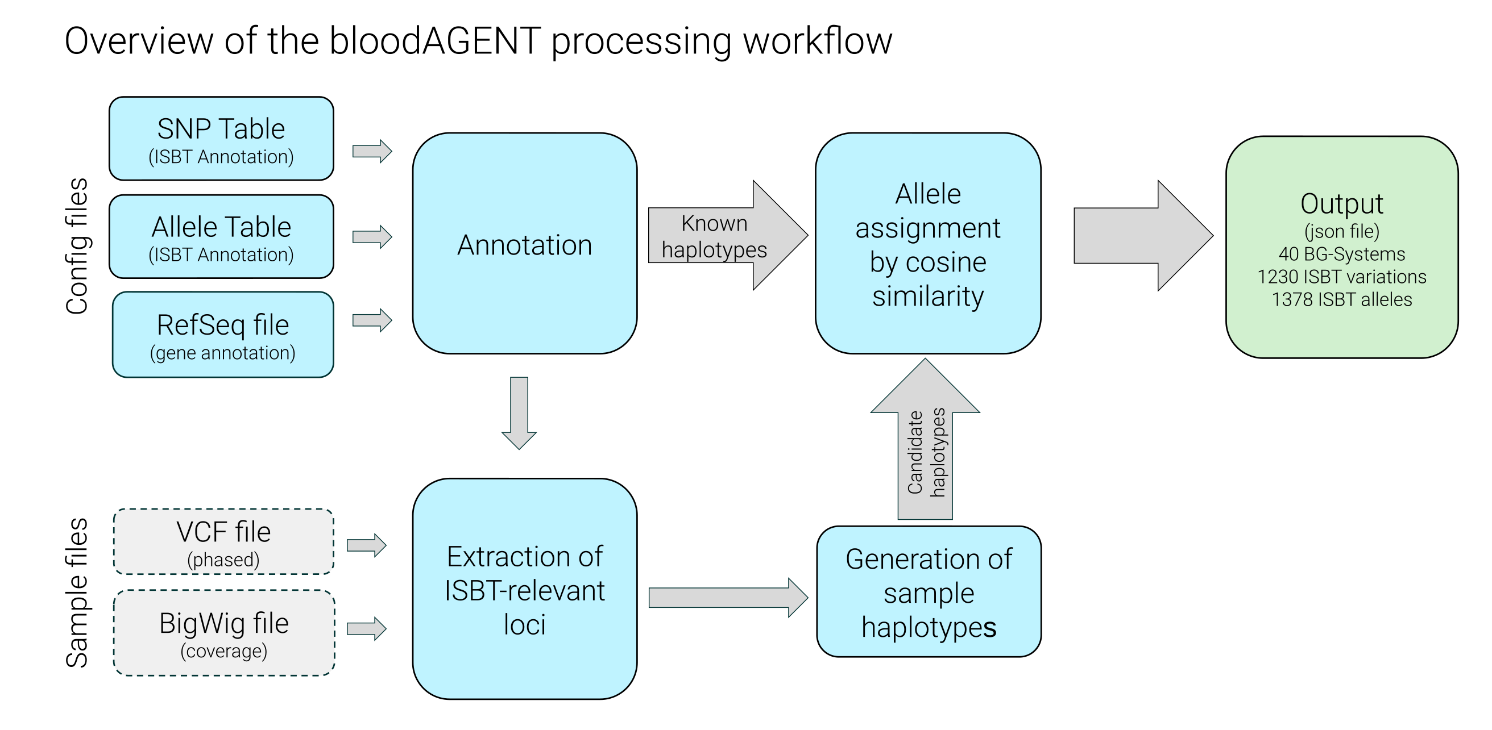
Supplementary Figure 1.** This diagram illustrates the modular structure and data flow of the bloodAGENT tool. The pipeline integrates phased genomic variant data and coverage information (VCF and BigWig files, respectively) with reference annotation sources, including an ISBT-curated SNP table, allele definitions, and RefSeq gene annotations. The process begins with the annotation of sample variants using ISBT-defined loci. Only variants relevant to blood group determination are retained, and used to construct phased haplotypes per sample. These haplotypes are then compared to known ISBT allele definitions using cosine similarity, enabling robust allele assignment even in the presence of incomplete variant patterns. The final output consists of a JSON-formatted report including predicted blood group alleles across 40 systems, incorporating 1230 ISBT-defined variants and 1378 alleles. The workflow emphasizes flexibility: all upstream preprocessing steps (alignment, variant calling, phasing) are external to bloodAGENT, allowing integration with custom or evolving pipelines. This modularity ensures that users can adapt the tool to various input formats and preprocessing standards.

**Supplementary Methods Table SM1.** Differences between haplotype calls from WhatsHap-phased and SHAPEIT5-phased datasets. Sample ID lists the sample identifier, and Locus indicates the blood group locus where the difference was detected. SHAPEIT5-exclusive genotypes contains allele combinations found only in SHAPEIT5-phased data, whereas WhatsHap-exclusive genotypes lists those found only in WhatsHap-phased data.An empty SHAPEIT5-exclusive genotypes cell indicates that bloodAGENT reported ambiguities for the WhatsHap-phased data but not for SHAPEIT5. Entries in both columns indicate conflicting results, usually at the sub-allele level. In each genotype, alleles 1 and 2 are separated by a comma and enclosed in quotation marks. Multiple alleles separated by a comma within the same quotation marks are equally likely for that position. For example, "ABO*O.01.01,ABO*O.01.02", "ABO*A1.01" indicates allele 1 is equally likely to be ABO*O.01.01 or ABO*O.01.02, while allele 2 is ABO*A1.01. Multiple possible genotypes are separated by semicolons.

| Sample ID | Locus | SHAPEIT5 exclusive genotypes | WhatsHap exclusive genotypes |
| --- | --- | --- | --- |
| HGDP00121 | ABO |  | "ABO*A1.01", "ABO*O.01.22" |
| HGDP00302 | ABO |  | "ABO*A1.01", "ABO*O.01.22" |
| HGDP00467 | ABO | "ABO*O.01.01", "ABO*O.01.09" | "ABO*O.01.09", "ABO*O.01.28" |
| HGDP00472 | ABO |  | "ABO*A1.01", "ABO*O.01.22" |
| HGDP00478 | ABO | "ABO*O.01.01", "ABO*O.01.02" | "ABO*O.01.02", "ABO*O.01.22,ABO*O.04.02,ABO*O.01.09" |
| HGDP00512 | ABO |  | "ABO*A1.01", "ABO*O.01.22" |
| HGDP00564 | ABO |  | "ABO*A1.01", "ABO*O.01.22" |
| HGDP00581 | ABO |  | "ABO*A1.02", "ABO*A2.06" |
| HGDP00590 | ABO |  | "ABO*A1.02", "ABO*O.01.29" |
| HGDP00630 | ABO | "ABO*O.01.01", "ABO*O.01.02" | "ABO*O.01.39,ABO*O.01.13", "ABO*O.01.83" |
| HGDP00640 | ABO |  | "ABO*A1.02", "ABO*A2.06" |
| HGDP00721 | ABO | "ABO*A2.09,ABO*cisAB.01", "ABO*O.01.02" | "ABO*A2.09", "ABO*O.01.02" |
| HGDP00730 | ABO |  | "ABO*A1.02", "ABO*O.01.29" |
| HGDP00757 | ABO | "ABO*A2.19", "ABO*B.01" | "ABO*A1.02", "ABO*B.01" |
| HGDP00806 | ABO |  | "ABO*A1.02", "ABO*A2.06" |
| HGDP00881 | ABO |  | "ABO*A1.01", "ABO*O.01.22" |
| HGDP00890 | ABO |  | "ABO*A1.02", "ABO*A2.06" |
| HGDP00900 | ABO |  | "ABO*A1.02", "ABO*A2.06" |
| HGDP00911 | ABO |  | "ABO*A2.16", "ABO*O.01.41" |
| HGDP00920 | ABO |  | "ABO*A1.02", "ABO*O.01.29" |
| HGDP00939 | ABO |  | "ABO*O.01.44", "ABO*O.01.83" |
| HGDP00944 | ABO | "ABO*A2.01", "ABO*O.01.02" | "ABO*A2.16", "ABO*O.01.23" |
| HGDP00985 | ABO |  | "ABO*A1.02", "ABO*O.01.29" |
| HGDP01151 | ABO |  | "ABO*A1.01", "ABO*O.01.22" |
| HGDP01255 | ABO |  | "ABO*A1.01", "ABO*O.01.22" |
| HGDP01264 | ABO |  | "ABO*A1.01", "ABO*O.01.22" |
| HGDP01360 | ABO |  | "ABO*A1.01", "ABO*O.01.22" |
| HGDP01397 | ABO |  | "ABO*A1.02", "ABO*A2.06" |
| HGDP01398 | ABO |  | "ABO*A1.01", "ABO*O.01.22" |
| HGDP01412 | ABO |  | "ABO*A2.16", "ABO*O.01.41" |
| HGDP00640 | DO | "DO*01", "DO*01" | "DO*01", "DO*01.-05" |
| HGDP00738 | DO | "DO*01", "DO*02" | "DO*01.-05", "DO*02" |
| HGDP00914 | DO | "DO*01", "DO*02.-04" | "DO*01.-05", "DO*02.-04" |
| HGDP00926 | DO | "DO*01", "DO*02" | "DO*01.-05", "DO*02" |
| HGDP00931 | DO | "DO*01", "DO*02" | "DO*01.-05", "DO*02" |
| HGDP00935 | DO | "DO*01", "DO*02" | "DO*01.-05", "DO*02" |
| HGDP00943 | DO | "DO*01", "DO*01" | "DO*01", "DO*01.-05" |
| HGDP01200 | DO | "DO*01", "DO*02" | "DO*01.-05", "DO*02" |
| HGDP01285 | DO | "DO*01", "DO*02" | "DO*01.-05", "DO*02" |
| HGDP01419 | DO | "DO*01", "DO*02.-04" | "DO*01.-05", "DO*02.-04" |
| HGDP00309 | FY |  | "FY*01,FY*01W.02", "FY*02" |
| HGDP00612 | FY |  | "FY*02", "FY*02N.01" |
| HGDP00624 | FY |  | "FY*02", "FY*02N.01" |
| HGDP00626 | FY |  | "FY*02", "FY*02N.01" |
| HGDP00646 | FY |  | "FY*01N.01", "FY*02" |
| HGDP00676 | FY |  | "FY*02", "FY*02N.01" |
| HGDP00686 | FY |  | "FY*01N.01", "FY*02" |
| HGDP00735 | FY |  | "FY*02", "FY*02N.01" |
| HGDP01257 | FY |  | "FY*02", "FY*02N.01" |
| HGDP00450 | GYPB | "GYPB*06.02", "GYPB*06.02" | "GYPB*03N.04", "GYPB*03N.04" |
| HGDP00458 | GYPB | "GYPB*04", "GYPB*06.02" | "GYPB*03N.03", "GYPB*06.01"; "GYPB*03N.04", "GYPB*04" |
| HGDP00462 | GYPB | "GYPB*06.02", "GYPB*06.02" | "GYPB*03N.04", "GYPB*03N.04" |
| HGDP00479 | GYPB | "GYPB*03", "GYPB*04" | "GYPB*03N.03", "GYPB*04" |
| HGDP00926 | GYPB | "GYPB*04", "GYPB*06.02" | "GYPB*03N.03", "GYPB*06.01"; "GYPB*03N.04", "GYPB*04" |
| HGDP00944 | GYPB | "GYPB*04", "GYPB*06.02" | "GYPB*03N.03", "GYPB*06.01"; "GYPB*03N.04", "GYPB*04" |
| HGDP00984 | GYPB | "GYPB*04", "GYPB*06.02" | "GYPB*03N.03", "GYPB*06.01"; "GYPB*03N.04", "GYPB*04" |
| HGDP00986 | GYPB | "GYPB*03", "GYPB*04" | "GYPB*03N.03", "GYPB*04" |
| HGDP01081 | GYPB | "GYPB*06.02", "GYPB*06.02" | "GYPB*03N.04", "GYPB*03N.04" |
| HGDP01086 | GYPB | "GYPB*03", "GYPB*04" | "GYPB*03N.03", "GYPB*04" |
| HGDP01201 | GYPB |  | "GYPB*03", "GYPB*06.01" |
| HGDP01285 | GYPB | "GYPB*04", "GYPB*06.02" | "GYPB*03N.03", "GYPB*06.01"; "GYPB*03N.04", "GYPB*04" |
| HGDP01418 | GYPB | "GYPB*03,GYPB*03N.01", "GYPB*06.01" | "GYPB*03N.01", "GYPB*06.01"; "GYPB*03N.02", "GYPB*04" |
| HGDP00047 | JK |  | "JK*01W.03", "JK*02,JK*01W.04"; "JK*01W.04", "JK*02,JK*01W.03" |
| HGDP00131 | JK | "JK*01", "JK*02,JK*01W.01" | "JK*01W.01", "JK*02" |
| HGDP00469 | JK | "JK*01", "JK*01W.03,JK*01W.04" | "JK*01W.03", "JK*01W.04" |
| HGDP00472 | JK | "JK*01", "JK*01W.03,JK*01W.04" | "JK*01W.03", "JK*01W.04" |
| HGDP00479 | JK | "JK*01", "JK*01W.03,JK*01W.04" | "JK*01W.03", "JK*01W.04" |
| HGDP00574 | JK |  | "JK*01W.03", "JK*02,JK*01W.04"; "JK*01W.04", "JK*02,JK*01W.03" |
| HGDP00620 | JK |  | "JK*01W.03", "JK*02,JK*01W.04"; "JK*01W.04", "JK*02,JK*01W.03" |
| HGDP00621 | JK |  | "JK*01W.03", "JK*02,JK*01W.04"; "JK*01W.04", "JK*02,JK*01W.03" |
| HGDP00624 | JK |  | "JK*01W.03", "JK*02,JK*01W.04"; "JK*01W.04", "JK*02,JK*01W.03" |
| HGDP00696 | JK |  | "JK*01W.03", "JK*02,JK*01W.04"; "JK*01W.04", "JK*02,JK*01W.03" |
| HGDP00726 | JK |  | "JK*01W.03", "JK*02,JK*01W.04"; "JK*01W.04", "JK*02,JK*01W.03" |
| HGDP00755 | JK |  | "JK*01", "JK*01W.01" |
| HGDP00813 | JK | "JK*01W.01,JK*01N.06", "JK*02" | "JK*01W.01", "JK*02N.01" |
| HGDP00818 | JK | "JK*01", "JK*02N.13,JK*02N.12,JK*02N.11" | "JK*01,JK*02N.13,JK*02N.12,JK*02N.11", "JK*02" |
| HGDP00904 | JK |  | "JK*01W.03", "JK*02,JK*01W.04"; "JK*01W.04", "JK*02,JK*01W.03" |
| HGDP00907 | JK | "JK*01", "JK*01W.03,JK*01W.04" | "JK*01W.03", "JK*01W.04" |
| HGDP00940 | JK | "JK*01", "JK*01W.03,JK*01W.04" | "JK*01W.03", "JK*01W.04" |
| HGDP00961 | JK |  | "JK*01", "JK*01W.01" |
| HGDP01280 | JK |  | "JK*01W.03", "JK*02,JK*01W.04"; "JK*01W.04", "JK*02,JK*01W.03" |
| HGDP01300 | JK |  | "JK*01W.03", "JK*02,JK*01W.04"; "JK*01W.04", "JK*02,JK*01W.03" |
| HGDP01405 | JK | "JK*01", "JK*01W.03,JK*01W.04" | "JK*01W.03", "JK*01W.04" |
| HGDP00205 | LU | "LU*02", "LU*02.19,LU*02.09" | "LU*02.09", "LU*02.19" |
| HGDP00458 | LU | "LU*01", "LU*02.19,LU*02.-24" | "LU*01.19", "LU*02.-24" |
| HGDP00462 | LU |  | "LU*01.19", "LU*02" |
| HGDP00601 | LU | "LU*02", "LU*02.-13" | "LU*02.-13", "LU*02.19"; "LU*02.-13", "LU*02.19,LU*02.-24" |
| HGDP00619 | LU | "LU*02", "LU*02.19,LU*02.-24" | "LU*02.-24", "LU*02.19" |
| HGDP00741 | LU |  | "LU*01", "LU*02.19" |
| HGDP00894 | LU | "LU*02", "LU*02.-13" | "LU*02.-13", "LU*02.-24" |
| HGDP00909 | LU |  | "LU*01", "LU*02.19" |
| HGDP00925 | LU | "LU*02", "LU*02.19,LU*02.-24" | "LU*02.-24", "LU*02.19" |
| HGDP00971 | LU | "LU*02", "LU*02.-13" | "LU*02.-13", "LU*02.-24" |
| HGDP01149 | LU |  | "LU*01", "LU*02.19" |
| HGDP01155 | LU |  | "LU*01", "LU*02.19" |
| HGDP01254 | LU | "LU*02", "LU*02.19,LU*02.-24" | "LU*02.-24", "LU*02.19" |
| HGDP01272 | LU |  | "LU*01.19", "LU*02" |
| HGDP01280 | LU |  | "LU*01.19", "LU*02" |
| HGDP01359 | LU |  | "LU*01.19", "LU*02" |
| HGDP01369 | LU | "LU*02", "LU*02.-13" | "LU*02.-13", "LU*02.19"; "LU*02.-13", "LU*02.19,LU*02.-24" |
| HGDP01372 | LU |  | "LU*01", "LU*02.19" |
| HGDP01412 | LU |  | "LU*01", "LU*02.19" |
| HGDP00546 | RHD | "RHD*01", "RHD*03.08" | "RHD*01W.40", "RHD*03.08"; "RHD*03.08", "RHD*37";  "RHD*03.08", "RHD*37,RHD*01W.40" |
| HGDP01086 | RHD | "RHD*09.02.01", "RHD*10.00" | "RHD*09.01.02", "RHD*10.00,RHD*05.04,RHD*10.05" |

References

Hofmeister, R.J., Ribeiro, D.M., Rubinacci, S. and Delaneau, O. (2023) Accurate rare variant phasing of whole-genome and whole-exome sequencing data in the UK Biobank. Nat. Genet., 55, 1243–1249. https://doi.org/10.1038/s41588-023-01426-9.
