# Supplementary material for: bloodAGENT: a versatile tool for blood group typing and genomic variation analysis
Source: Bioinform Adv. 2025 Sep 2;5(1):vbaf210. doi: 10.1093/bioadv/vbaf210 (PMC12448795; doi:10.1093/bioadv/vbaf210)
Supplement: vbaf210_Supplementary_Data [file vbaf210_supplementary_data.zip › bloodAgent_Application_Note_Supplementary_Figure_1.docx]

| Supplementary Figure 1 of  bloodAGENT: A Versatile Tool for Blood Group Typing and Genomic Variation Analysis  Michael Wittig^1,*^, Tim A. Steiert^1^, Christoph Gassner^1,2^, Andre Franke^1^  ^1^ Institute of Clinical Molecular Biology, Christian-Albrechts-University and University Medical Center Schleswig-Holstein, Kiel 24105, Germany  ^2^ Institute of Translational Medicine, Private University in the Principality of Liechtenstein, Triesen 9495, Liechtenstein  *Corresponding author. Zentrum Molekulare Biowissenschaften, Am Botanischen Garten 11 – 24118 Kiel, Germany. E-mail:m.wittig@ikmb.uni-kiel.de |
| --- |


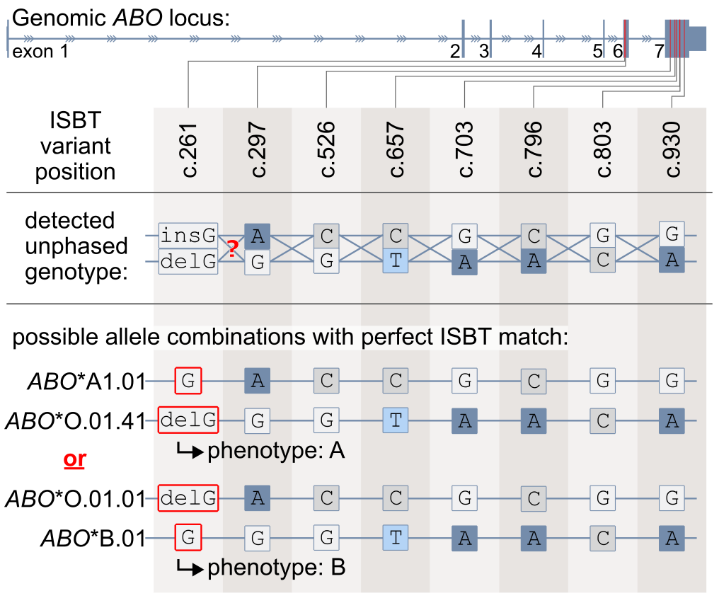


**Supplementary Figure 1.** Ambiguities in the *ABO* blood group system. Genetic variants in the *ABO* locus are used to determine the reference allele. In cases where genotyping data lack haplotype phasing, certain ISBT coding variants can be recombined in two ways, both aligning with the reference allele, leading to ambiguity in allele assignments. This is illustrated for the *ABO**A1.01/*ABO**O.01.41 and *ABO**O.01.01/*ABO**B.01 allele combinations, where the respective indel (“G”/”delG”) at position c.261, in conjunction with the remaining variants, determines the correct allele call. This ambiguity is particularly critical, as it affects phenotype determination, resulting in A and B antigens in the respective cases, potentially leading to severe immunohematological reactions.
